# Co-occurrence of m.1555A>G and m.11778G>A mitochondrial DNA mutations in two Indian families with strikingly different clinical penetrance of Leber hereditary optic neuropathy

**Published:** 2013-06-11

**Authors:** Nahid Akhtar Khan, Periyasamy Govindaraj, Vuskamalla Jyothi, Angamuthu K Meena, Kumarasamy Thangaraj

**Affiliations:** 1 CSIR-Centre for Cellular and Molecular Biology, Hyderabad, India; 2 Department of Neurology, Nizam’s Institute of Medical Sciences, Hyderabad, India

## Abstract

**Background:**

Mitochondrial DNA (mtDNA) mutations are known to cause Leber hereditary optic neuropathy (LHON). However, the co-occurrence of double pathogenic mutations with different pathological significance in pedigrees is a rare event.

**Methods:**

Detailed clinical investigation and complete mtDNA sequencing analysis was performed for two Indian families with LHON. The haplogroup was constructed based on evolutionarily important mtDNA variants.

**Results:**

We observed the existence of double pathogenic mutations (m.11778G>A and m.1555A>G) in two Indian LHON families, who are from different haplogroup backgrounds (M5a and U2e1), with different clinical penetrance of the disease (visual impairment). The m.11778G>A mutation in the *MT-ND4* gene is associated primarily with LHON; whereas, m.1555A>G in the *12S rRNA* gene has been reported with aminoglycoside-induced non-syndromic hearing loss.

**Conclusions:**

The absence of hearing abnormality and widely varying clinical expression of LHON suggest additional nuclear modifier genes, environmental factors, and population heterogeneity might play an important role in the expression of visual impairment in these families.

## Introduction

Leber hereditary optic neuropathy (LHON; OMIM- Online Mendelian Inheritance in Man 535000) was the first disease associated with mitochondrial DNA (mtDNA) point mutation [1]. LHON is mainly characterized by maternally inherited painless loss of central vision, predominantly affecting males [2,3]. More than 90% of LHON cases across the world are due to three primary mutations (m.3460G>A in *MT-ND1*, m.11778G>A in *MT-ND4*, and m.14484T>C in *MT-ND6*) in mtDNA genes, encoding different subunits of complex I [2,4]. These mutations impair the function of respiratory chain complex I, although the exact mechanism elucidating their functional significance is not fully understood [5,6]. However, not all individuals who inherit these primary mtDNA mutations develop optic neuropathy and visual impairment, which shows the variable degree of penetrance among different pedigrees [4,7]. Incomplete penetrance along with sex bias suggests the possibility of other genetic and environmental factors involved in the etiology of the disease expression [8-11].

More than 300 mtDNA mutations to date have been reported to be associated with different diseases. Among many disease-causing mtDNA mutations, m.1555A>G in the *12S rRNA* gene has been confirmed to cause aminoglycoside-induced non-syndromic hearing loss [12,13]. The coexistence of more than one disease causing primary mtDNA mutations in pedigrees remains a rare occurrence. To date, a single pedigree from China has been reported with m.11778G>A and m.1555A>G mutations with high clinical penetrance of LHON, suggesting their synergistic role in disease penetrance [14], while another pedigree has m.14484T>C and m.1555A>G mutations with different clinical penetrance and hearing impairment [15].

Here, we report two unrelated pedigrees from India with m.11778G>A and m.1555A>G mutations with varying clinical expression.

## Methods

### Clinical evaluation and sample collection

Of the 187 families with LHON referred to our center for the genetic study, we selected two families for the mtDNA study; one is Dravidian, and the other is Indo-European. The patients were physically examined by ophthalmologists and clinicians at Nizam’s Institute of Medical Sciences (NIMS), Hyderabad. The major clinical diagnosis of the patients with LHON was due to acute or subacute, sudden, painless, central vision loss leading to central scotoma and dyschromatopsia. Ophthalmologic examinations, including visual acuity and visual field examination, of the probands and other members of these families were conducted. The degree of visual impairment was defined according to the visual acuity as follows: normal >0.3, mild=0.3–0.1, moderate <0.1–0.05, and severe <0.05–0.02. Informed written consent was obtained from the individuals who participated in this study. The study adhered to the tenets of the Declaration of Helsinki and was approved by the Institutional Ethical Committees (IECs) of all the participating institutions.

### Genetic analysis

DNA was extracted from blood samples using a standard protocol [16]. The complete mtDNA of the proband samples was amplified using 24 sets of primers to generate overlapping fragments [17,18]. The overlapping amplicons were directly sequenced (forward and reverse, separately) using the ABI BigDye Terminator Cycle Sequencing kit (Applied Biosystems, Foster City, CA). Extended products were precipitated with ethanol: sodium acetate, washed with 70% alcohol, dried, then dissolved in Hi-Di formamide, and analyzed in the ABI 3730 DNA Analyzer (Applied Biosystems). All 48 (24 forward and 24 reverse) sequences were edited using sequence analysis and assembled with the revised Cambridge Reference Sequence (rCRS; NC_012920) [19,20] using AutoAssembler software (Applied Biosystems). The observed mutations were compared with mitochondrial databases such as Mitomap, mtDB, and HmtDB for their significance. The mtDNA haplogroup was assigned to each individual based on the mutation, using the available literature (Phylotree; mtDNA tree Build 15 (30 Sep 2012). In addition, we also assessed the m.11778G>A and m.1555A>G mutations in available family members using restriction fragment length polymorphisms. The m.11778G>A mutation was analyzed as described earlier [21], and m.1555A>G was amplified using the primer (forward: 5′-CCC CAG AAA ACT ACG ATA GCC-3′ and reverse: 5′-ACA CTC TGG TTC GTC CAA GTG-3′). The amplified product was digested using restriction enzyme (BsmAI) and analyzed with electrophoresis in a 1.5% agarose gel stained with ethidium bromide. The sequence generated in this study has been submitted to the GenBank database (accession number JX462687 and JX462739).

## Results

### Clinical features

**Figure 1:**
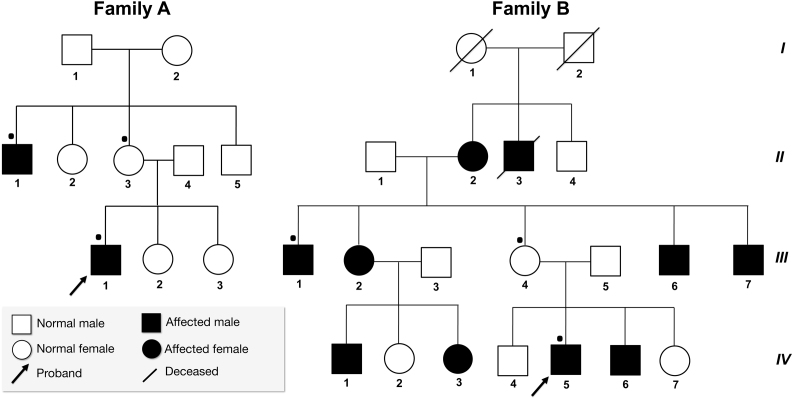
Pedigree information of two Indian families (**A**, **B**) with Leber hereditary optic neuropathy with mutations m.11778G>A in *MT-ND4* gene and m.1555A>G in *12S rRNA*. Samples analyzed are denoted by dot (•).

Family A (P23) is from Andhra Pradesh, in southern India, and family B (P75) is from Punjab, in northern India. The average age of onset of LHON in family A was 19.5 years while for family B the average was 23.6 years. The overall penetrance of LHON in both families was strikingly different. The penetrance of family A was 25%, with two out of eight individuals affected (Figure 1). Family B presented with high clinical penetrance; that is, 62.5% with ten out of sixteen individuals affected (Figure 1). None of the members from either family reported a hearing abnormality. In family A, 50% (2/4) of the men were affected, and none of the women had a history of visual impairment, while in family B, men (7/10) and women (3/6) were affected. Clinical manifestations of the patients are given in Table 1. None of the patients reported a change in their dietary intake or exposure to drugs or toxic agents around the time of visual loss. Detailed clinical analysis and previous records of the families did not reveal any other abnormalities such as neurological disorders, hearing impairment, muscular diseases or exercise intolerance, etc., which are mostly associated with mitochondrial dysfunctions. After the m.1555A>G mutation was confirmed in both families, the patients were subjected to audiological examination. Neurologic examination of the 8th nerve as demonstrated by Webers and absolute bone conduction test was normal. Brain stem auditory potential and audiogram were also within normal range (10 to 25 dB). The patients did not have specific records regarding exposure to specific antibiotics that have a significant role in aminoglycoside-induced hearing impairment.

**Table1:** Clinical features of family A and family B.

Family	Patient ID	Age of onset (yrs)	Sex	Visual Acuity		Visual Fields		Fundus finding
				OD	OS	OD	OS	
Family A	II:1	22	M	0.02	0.01	Central cecal scotoma	Central scotoma	Diffuse disc polar
	III:1	17	M	0.1	0.08	Central scotoma	Central scotoma	Diffuse disc polar
Family B	II:2	-	F	-	-	ND	ND	ND
	II:3	-	M	-	-	ND	ND	ND
	III:1	23	M	0.01	0.01	Central scotoma	Central scotoma	Diffuse disc polar
	III:2	26	F	0.1	0.04	Central scotoma	Central scotoma	Diffuse disc polar
	III:6	21	M	0.03	0.04	Central cecal scotoma	Central scotoma	Diffuse disc polar
	III:7	24	M	0.02	0.02	Central scotoma	Central scotoma	Diffuse disc polar
	IV:1	25	M	0.04	0.08	Central cecal scotoma	Central cecal scotoma	Diffuse disc polar
	IV:2	26	F	0.1	0.05	Central scotoma	Central scotoma	Diffuse disc polar
	IV:5	23	M	0.05	0.05	Central scotoma	Central scotoma	Diffuse disc polar
	IV:6	21	M	0.2	0.1	Normal	Central cecal scotoma	Diffuse disc polar

Abbreviations: OD represents right eye; OS represents left eye; ND not done

### Mitochondrial DNA mutation analysis

**Figure 2:**
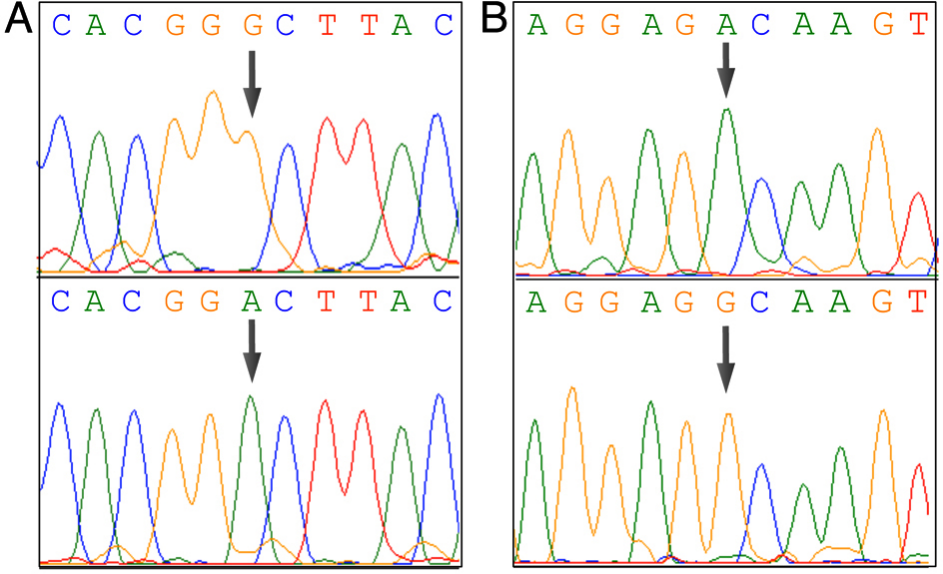
Sequence electropherograms of mitochondrial DNA mutations. **A**: The upper panel shows (arrow) the wild-type nucleotide G at the position 11,778 in *MT-ND4* gene, while the lower panel shows the mutant allele A that changes the amino acid from alanine to valine. **B**: The upper panel shows (arrow) the wild-type nucleotide A at the position 1555 in *12S rRNA* gene, while the lower panel shows the mutant allele G.

After complete clinical assessment, the probands and available family members were screened for primary LHON mutations (m.3460G>A, m.11778G>A, and m.14484T>C). One of the primary LHON mutations (m.11778G>A in the *MT-ND4* subunit of complex I) was found (Figure 2 and Appendix 1). The analysis of complete mtDNA revealed a total of 59 variations, including the m.1555A>G mutation in both pedigrees, which is highly conserved in the *12S rRNA* gene (Figure 2 and Appendix 1). These mutations (m.11778G>A and m.1555A>G) were present in homoplasmic state in the probands and other family members; thus, heteroplasmy could not be the reason for the clinical variability (Appendix 1).

**Figure 3:**
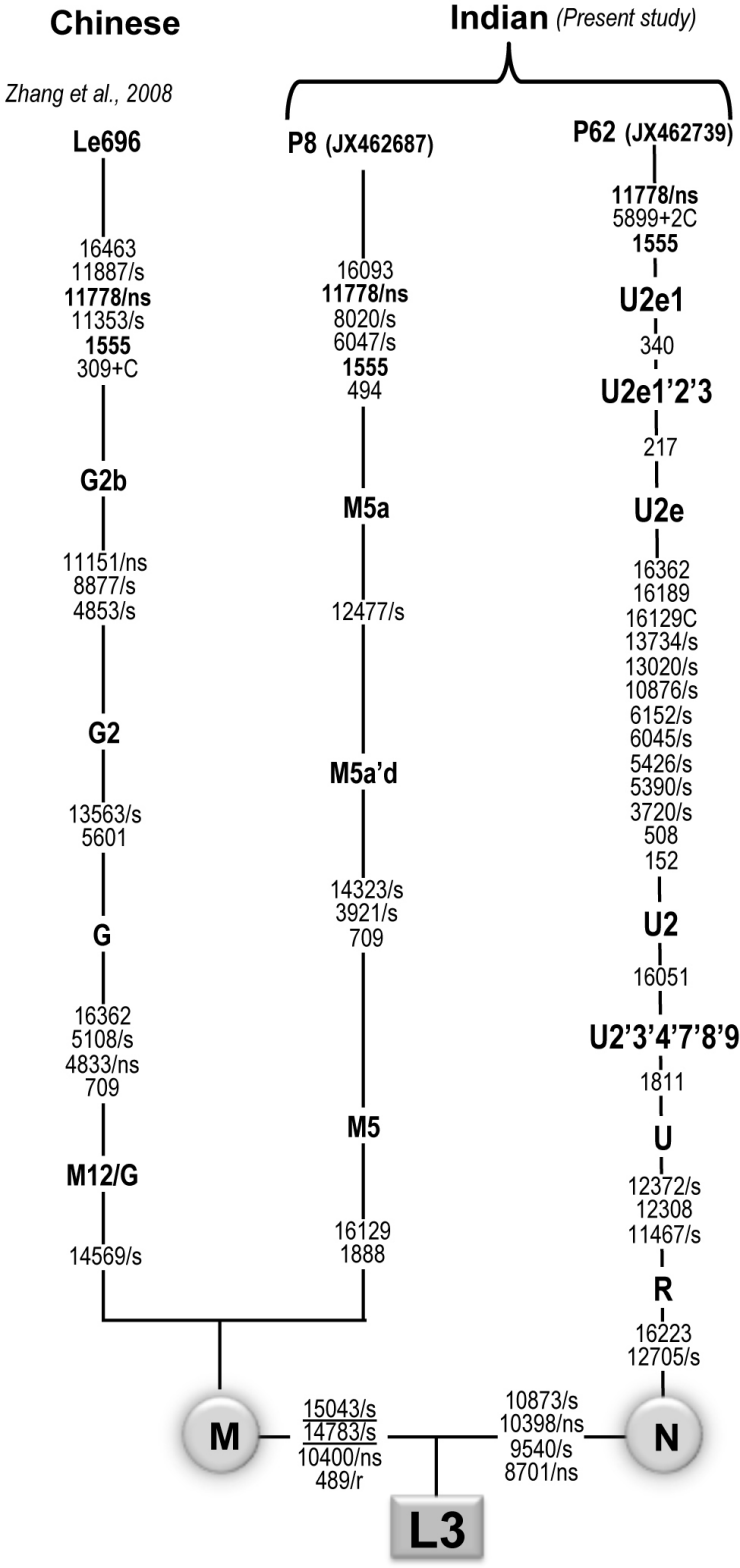
Comparison of the haplogroup of two Indian families with the Chinese sample carrying double mutations (m.117788G>A and m.1555A>G). The synonymous and non-synonymous are denoted by “s” and “ns,” respectively, rRNA genes by “r,” and the underlined mutations are recurrent. Zhang et al. (2008) refers to [14].

Haplogroups constructed based on the mtDNA sequence suggested that family A belongs to the M5a haplogroup while family B belongs to the U2e1 haplogroup (Figure 3). Both families belonged to different ethnic and linguistic affiliations in India. Family A lives in southern India and speaks the Dravidian language; family B lives in northern India and speaks an Indo-European language. Among all the variants observed in both families, only m.11778G>A and m.1555A>G were confirmed disease-causing mutations. In addition to haplogroup-defining motifs, family A also showed two private mutations, m.6047G>A and m.8020G>A. Neither variant is present in the M5a haplogroup motif. The complete mtDNA sequence variations of both families are shown in Appendix 2 and Appendix 3.

## Discussion

In the present study, clinical, genetic, and molecular characterization of two Indian families with LHON was performed. Bilateral visual impairment in the family members strongly suggested mitochondrial involvement. After initial diagnosis, complete mtDNA sequencing revealed the coexistence of double pathogenic mutations (m.11778G>A and m.1555A>G) in the probands and their matrilineal relatives. This is the first time these two mutations have been observed in Indian families. Two earlier reports from China found the coexistence of two pathogenic mutations: one study with m.11778G>A and m.1555A>G [14] and other with T14484C and m.11778G>A [15]. Both earlier reports showed variable clinical penetrance and disease severity in Chinese families.

In the present study, none of the maternally related members from either family have a hearing impairment, which is similar to an earlier report by Zhang et al. (2008), whereas in the family reported by Wei et al. (2007), one out of 14 maternal relatives exhibited mild hearing loss in addition to LHON. Both families in the present study showed incomplete clinical penetrance, whereas the visual impairment is higher in family B (ten out of sixteen; 62.5%) compared to family A (two out of eight; 25%). The average age of onset in families A and B was 19.5 and 23.6 years, respectively. However, this difference could be due to the difference in the number of affected individuals. The presence of visual impairment only and the absence of hearing abnormality in both families suggests that the presence of two pathogenic mutations may not necessarily result in expression of their independent pathologies.

Incomplete penetrance, variable age of onset, and sex bias have been observed in LHON pedigrees [7,22,23] as we also observed in the present study. This suggests the role of other modifier factors such as nuclear gene(s) background, environmental factors, and mitochondrial variants/haplotypes in the phenotypic manifestation of the m.11778G>A mutation. In particular, the mitochondrial variants/haplotypes have been shown to influence the penetrance and expressivity of vision loss associated with the primary mtDNA mutations [4,7]. Earlier studies suggested that the J/T haplogroup-defining variants m.4216T>C and m.13708G>A increase the penetrance and expressivity of LHON with the primary mutations, m.11778G>A and m.14484T>C [24]. Furthermore, other variants such as m.7444G>A in *MT-COI*, m.4435A>G in tRNA*^Met^*, m.15951A>G in tRNA*^Thr^* and m.11696G>A in *MT-ND4* mutations have potential roles in increasing the penetrance and expressivity of primary LHON associated with the m.11778G>A mutation [25-27]. Of the variants observed in both families, the m.1555A>G homoplasmic mutation in the *12S rRNA* gene has been associated with aminoglycoside-induced and non-syndromic deafness in many families worldwide [12,13]. Functional characterization of cell lines harboring the m.1555A>G mutation showed mitochondrial dysfunction and sensitivity to aminoglycosides in the maternal relatives of a large Arab-Israeli family [28]. Therefore, it makes sense that the presence of m.1555A>G in the present study might have a synergistic effect on primary LHON mutation. Based on an earlier report by Zhang et al. [14] and seeing the high penetrance in family B, we cannot rule out this possibility. However, family A showed comparatively lower penetrance and thus contradicts the fact that the presence of m.1555A>G is the only factor responsible for the increase in clinical penetrance. Similar to primary LHON mutations, phenotypic expression of m.1555A>G has also been shown to be modulated by other factors such as nuclear gene(s) and environment factors, and thus could be one of the reasons for the absence of hearing loss in both families. Evolutionary analysis suggests that these two families belonged to different haplogroups (M5a and U2e1). Both haplogroups are identified by several different motifs. This suggests that these mutations occurred independently against different haplogroup backgrounds in the Indian population. Considering the heterogeneity of the Indian population, due to the practice of strict endogamy marriage over the past thousands of years, the multiple occurrence of the primary mutations (m.11778G>A and m.1555A>G) in different haplogroup, ethnic, and linguistic backgrounds is not surprising.

In conclusion, we report for the first time the co-occurrence of two pathogenic mutations, m.11778G>A and m.1555A>G, in two Indian families with different haplogroup, ethnic, and linguistic backgrounds. Both families show strikingly different clinical expression suggesting additional nuclear modifier genes and environmental factors play an important role in the expression of visual impairment in LHON. However, a long-term follow-up study will provide more information about the disease expression in these families.

## Appendix 1. Detection of m.11778G>A and m.1555A>G mutations using PCR-RFLP.

A) The mutation m.11778G>A was analyzed as described earlier [21], the gel picture showing wild-type allele (G; 265 bp) and mutant (A; 217 bp). B) The gel picture of m.1555A>G mutation showing wild-type allele (A; 182 bp) and mutant (G235 bp). To access data click "Appendix 1".

## Appendix 2. List of complete mtDNA variants observed in Family A.

To access data click "Appendix 2".

## Appendix 3. List of complete mtDNA variants observed in Family B.

To access data click "Appendix 3".
